# An inappropriate pacing threshold increase after repeated electrical storm in a patient with implantable cardioverter defibrillator

**DOI:** 10.1186/s12872-017-0695-y

**Published:** 2017-10-16

**Authors:** Ye Zhu, Xiang Gu, Chao Xu

**Affiliations:** 1 Clinical Medical College, Yangzhou University, Yangzhou, Jiangsu 225001 China; 2 Department of Cardiology, Northern Jiangsu Province Hospital, Yangzhou, Jiangsu 225001 China; 3 Department of Global Biostatistics and Data Science, School of Public Health and Tropical Medicine, Tulane University, New Orleans, LA 70112 USA

**Keywords:** Implantable cardioverter defibrillator, Anti-tachycardia pacing, Pacing threshold, Electrical storm

## Abstract

**Background:**

Implantable cardioverter defibrillators (ICD) are capable of effectively terminating malignant ventricular arrhythmia and are the most effective way to prevent sudden cardiac death. However, some evidences demonstrated that both anti-tachycardia pacing (ATP) and ICD shock can also bring adverse prognosis.

**Case presentation:**

A 66-year-old Han Chinese man with prior ICD implantation was admitted to our hospital because of frequent ICD shocks. Although intravenous amiodarone and esmolol succinate were administered daily, the patient suffered 155 episodes of VT/VF during 8 weeks after implantation. After repeated discharge of the device, the pacing threshold of the patient increased gradually. Considering the inappropriate increase of the pacing threshold, we decided to reposition the right ventricular (RV) lead with good sensing and threshold parameters confirmed. Subsequent 22 months interrogation follow-up revealed a stable lead position and electrical specifications. Furthermore, antiarrhythmic drugs were maximally increased, while ATP burst was remarkably decreased and the inappropriate ICD shock never occurred until now.

**Conclusion:**

An inappropriate pacing threshold was increased secondary to repeated ICD electrical storm. A timely active lead position adjustment reduced the pacing threshold and eliminated the risk of premature battery depletion.

**Electronic supplementary material:**

The online version of this article (10.1186/s12872-017-0695-y) contains supplementary material, which is available to authorized users.

## Background

Implantable cardioverter defibrillator (ICD) can effectively terminate malignant ventricular tachycardia (VT)/ventricular fibrillation (VF) and prevent sudden death [1–3]. Electrical storm (ES), characterized by multiple attacks of VT or ventricular fibrillation (VF), is an unstable condition for which management and prevention are still a challenge. Whether ES is a causal factor or only an epiphenomenon remains unclear, although it is still a matter for debate that repeated shocks may provoke myocardial damage and result in further deterioration of the underlying disorders [4].

We report an ICD patient with severe ES caused by frequent VT/VF refractory to antiarrhythmic agents. The continuous discharge of the ICD might be related to an inappropriate increase of pacing threshold and it necessitated a repositioning of a lead.

## Case presentation

**Fig. 1:**
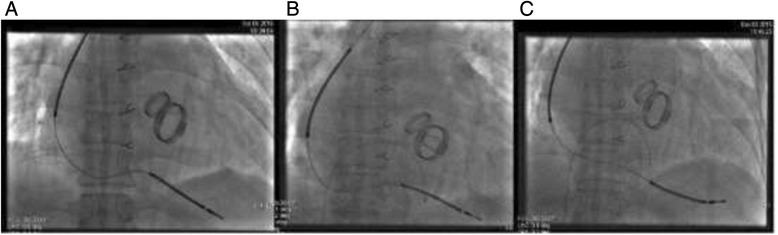
Anteroposterior chest X-ray follow-up of the ICD lead position. **a** ICD lead position after first implant. **b** No apparent dislocation of the lead at 9 weeks post- first implant. **c** Chest X-ray image of lead reposition. ICD, implantable cardioverter-defibrillators

A 66-year-old Han Chinese male was admitted after several VT attacks over the course of 1 month. The patient was diagnosed with rheumatic heart disease and impaired left ventricular ejection fraction (LVEF) of 42% in a functional New York Heart Association stage III, suffering from VT/VF and atrial fibrillation. He underwent surgery of the mitral valve and an aortic valve replacement 10 years ago. Electrolytic measurements showed sodium, calcium, and potassium values were within the normal range. He was treated with aldosterone antagonist, angiotensin converting enzyme inhibitor, lidocaine, β-blocker, and amiodarone, but VT recurred despite the optimized drug treatment. Additional doses of antiarrhythmic drugs were administered but unsuccessful. The patient was placed on amiodarone 200 mg orally once a day and metoprolol 25 mg orally four times a day. Attempts to reduce the frequency of VT using optimized antiarrhythmic drugs failed. VT could only be terminated by external cardioversion. The patient satisfied the requirement for ICD therapy according to the criteria of the Sudden Cardiac Death in Heart Failure Trial (SCD-HeFT), [5] so a single chamber ICD (Model 1231–40, St. Jude Medical Cardiac Rhythm Management, USA) was implanted with lead in the right ventricular (RV) apex after symptom stabilization (Fig. 1a). During the implantation, electrical specifications were confirmed, including the R-wave of 9.0 mV, the slew rate of 1.7 V/s, the impedance of 574 Ohm, and pacing threshold of 0.6 V/0.5 ms.

**Fig. 2:**
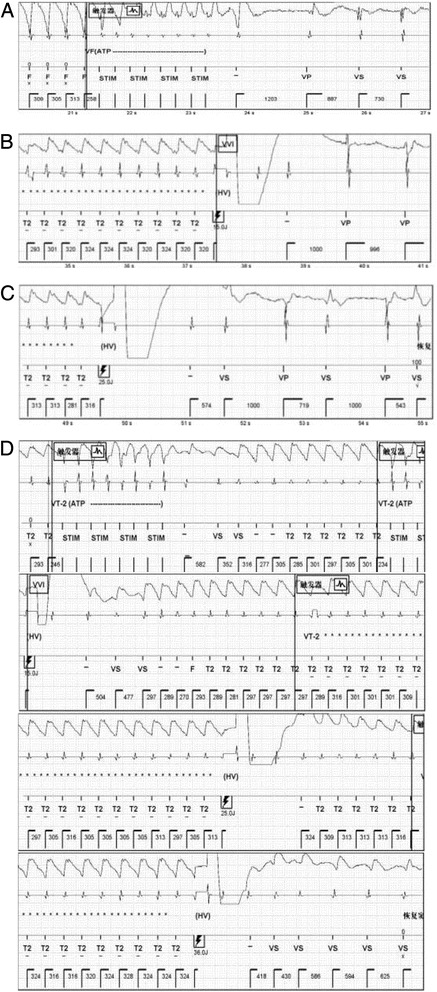
ICD interrogation record of VT/VF and cardioversion. **a**, **b** and **c** representing a successful ATP burst, a 15.0 J shock and a 25.0 J shock cardioversion respectively. **d** A 36.0 J shock terminated VT after No successful ATP burst, a 15.0 J shock and a 25.0 J shock cardioversion. ICD, implantable cardioverter-defibrillators; VT, ventricular tachycardia; VF, ventricular fibrillation; ATP, anti-tachycardia pacing

Two days later, the patient was abruptly awakened by an episode of VT/VF. The initial administration of the ATP therapy through the ICD was unsuccessful. VF was finally terminated by a 36 J ICD shock. Frequent ICD shocks were associated with anxiety and intolerance of ICD therapy. The patient was thin and weak. ATP and Low energy cardioversion (15/25 J) were successfully used to relieve the patient’s pain, anxiety and possible myocardial injury (Fig. 2-a, b, c). Overall, the patient experienced 155 episodes of VT/VF and received a total of 27 shocks after the first implantation of ICD. The most severe attack was 34 VT/VF episodes within 24 h. Rarely, a 36.0 J shock at last reverted VT, which was not terminated by ATP burst and a 15.0 J/25 J shock (Fig. 2-d). Frequent ICD discharges depleted the battery life considerably. His treatment was aggressively escalated. Catheter ablation was contraindicated because of the patient’s metal valve and multiple original VTs. Renal artery denervation was recommended but refused. Attempts to reduce the frequency of VT only using antiarrhythmic drugs, such as lidocaine, were unsuccessful. The combination of ATP and intravenous esmolol seemed to reduce, but did not fully eliminate the episodes of VT.

After repeated discharge of the device for 8 weeks, the capture threshold was gradually increased. Initial threshold of device interrogation was respectively 2.37 V/0.5 ms, 2.75 V/0.5 ms, and 3.75 V/1.0 ms in 2, 4 and 9 weeks post- implantation (Additional file 1). Thorax X-ray at 9 weeks post-implant did not show apparent dislocation of the lead (Fig. 1b). Considering the patient’s reliance on the pacemaker, we decided to reposition the RV lead to better match the ventricular myocardium in the threshold field as a final alternative (Fig. 1c). Fortunately, this remedy did lower the pacing threshold. Lead measurements showed the ventricular sensing at 11.3 mV, a pacing threshold of 1.4 V/0.5 ms, and an impedance of 730 Ohms. The further clinical course of the patient was uncomplicated and he was discharged in good clinical status after the surgery. Recent device interrogation respectively showed a ventricular sensing of 8.9 mV, 7.9 mV and 9.1 mV, a pacing threshold of 1.75 V/0.5 ms, 1.5 V/1.0 ms and 1.5 V/1.0 ms at 13, 20, 24 months post-implantation follow-up (Additional file 1). Amiodarone 200 mg qd and metoprolol 25 mg q6h were given to enhance the efficacy of anti-arrhythmias. The patient’s pacing threshold and ventricular arrhythmias have carefully being monitored in the long term. Until now, the shock never occurred again and ATP burst significantly decreased.

## Discussion and conclusions

This case highlights a rare clinical situation: an increased pacing threshold may be increased by repeated ES in a patient with an ICD.

In the era of ICD therapy, ES has become increasingly common. It is generally defined as the occurrence of three or more obvious episodes of VT /VF in 24 h, separated by bouts of normal rhythm after successful therapy, either ATP burst or shock [6]. Previous studies have shown that ES occurs in about 10 – 28% of ICD recipients and is associated with increased mortality [7]. The first therapy for ES is eliminating the risk factors as soon as possible, then following a positive comprehensive management protocol [8]. Structural heart disease, electrolyte imbalance, myocardial infarction, inherited arrhythmic syndrome and other factors could all lead to ES [9]. In this case, VT was definitively identified on admission. The patient had a history of heart failure, rheumatic heart disease, and valve replacement surgery. He did not have diabetes, hyperlipidemia, a history of smoking or any other risk factors for coronary heart disease. Risk factors for acidosis and electrolyte disorders were also excluded [10]. The patient had considerable VTs during the perioperative period of ICD implantation. ES before ICD implantation may cause myocardial damage, and the placement of the ICD electrode may further damaged the local myocardium. The inappropriate discharge during 8 weeks after ICD implantation was attributed to myocardial injury caused by ES before ICD implantation. Additionally, the occurrence of ES seriously affected the patient’s quality of life and induced psychological symptoms including anxiety and fear. This programming strategy should be minimal energy of the defibrillation and effectively terminate VT/VF. However, some patients at high risk for sudden cardiac death still require the maximum energy that ICDs can achieve. For such patients, a readjustment of the RV lead position is a successful strategy to lower the threshold [11].

Overdrive pacing, as implemented in this case, has been reported to stabilize an ES [12]. Comprehensive treatment of VT such as increasing the identifying frequency of VT, optimal medication, trigger or substrate ablation and denervation should be administered to reduce or eliminate false identification and inappropriate ICD discharges [13]. As illustrated in this case, the management of ES hinges on excluding every possible causative factor, no matter how rare. Catheter ablation appears to be effective in both short-term therapy and prevention of ES. Catheter ablation of the left ventricular was contraindicated because of the patient’s metal valve. Renal artery denervation was offered but refused. Amiodarone and beta­blockers might lower the incidence of shock therapy in patients with ICD. Optimal programming aimed at reducing the burden of ICD therapy without an increase in adverse outcomes [14]. For this case, post-implantation inspection showed that the pacing threshold gradually increased. Thorax X-ray at 9 weeks post-implant did not show apparent dislocation of the lead. However, a lead tiny dislodgement seldom be excluded or confirmed. The repeated ES may have facilitated the development of heart failure and myocardium injury, which in turn increased the pacing threshold and shortened the life of the device. We took into consideration the fact that RV lead repositioning is a surgical procedure that exposes the patient to several risks. RV-ICD lead position adjustment can also be successfully performed to avoid the pacing threshold increase. Low pacing threshold is not only critical for the safety and effectiveness of the ICD therapy, but also can extend the life of the device. We made several efforts, invasively and noninvasively, to decrease this patient’s pacing threshold, including lead repositioning and appropriate ICD adjustment [15]. Here, the positioning of the RV lead was used as a final remedy. The 22 months follow-up after the second ICD implantation showed a steady position of the RV lead and stable parameters for sensing and pacing. The inappropriate shock never occurred until now. However, lead repositioning may not lead to ES cessation. ES cessation was achieved by adjusting the administration and dosage of antiarrhythmic agents and myocardium rehabilitation.

In this case, the attack of VT/VF was safely terminated by maximal post-procedural administration of antiarrhythmic drugs, subsequent application of ATP and discharge by the ICD. The follow-up showed that an inappropriate pacing threshold by repeated ES could not be fully eliminated. A timely active lead position adjustment reduced the pacing threshold and eliminated the risk of premature battery depletion. A programmable strategy should be personally adjusted as the low, middle and high cardioversion energy for effectively terminating VT/VF and lessening myocardial injury secondary to ES.

## Additional file

Additional file 1: I. 14 and 28 Oct, 2015 in 4 weeks post-first implantation follow-up. II.16, 18, 22, 24 and 30 Nov, 2015 in 2 months post- first implantation follow-up. III.1 and 3 Dec, 2015 in 2 months post- first implantation follow-up. IV.8, 9 and 16 Dec, 2015 in two weeks post- second implantation follow-up. V. 9 Mar and 13 Apr, 2016 in 3–4 months post- second implantation follow-up. VI. 9 Nov, 2016 in 9 months post- second implantation follow-up. VII. 14 Jun, 2017 in 18 months post- second implantation follow-up. VIII. 27 Sep, 2017 in 22 months post- second implantation follow-up. Initial threshold of device interrogation was respectively 2.37 V/0.5 ms, 2.75 V/0.5 ms, and 3.75 V/1.0 ms in 2, 4 and 9 weeks post- implantation. Recent device interrogation showed a ventricular sensing of 8.9 mV, 7.9 mV and 9.1 mV, a pacing threshold of 1.75 V/0.5 ms, 1.5 V/1.0 ms and 1.5 V/1.0 ms at 13, 20, 24 months post-implantation follow-up. (DOCX 902 kb)

## Abbreviations

ATP: Anti-tachycardia pacing
ECG: Electrocardiogram
ES: Electrical storm
ICD: Implantable cardioverter-defibrillators
LVEF: Left ventricular ejection fraction
RV: Right ventricular
VF: Ventricular fibrillation
VT: Ventricular tachycardia
