# Supplementary material for: An inappropriate pacing threshold increase after repeated electrical storm in a patient with implantable cardioverter defibrillator
Source: BMC Cardiovasc Disord. 2017 Oct 16;17:265. doi: 10.1186/s12872-017-0695-y (PMC5644131; doi:10.1186/s12872-017-0695-y)

**Additional file 1**

**Device Interrogation follow-up data**

I. 14 and 28 Oct, 2015 in 4weeks post-first implant follow-up


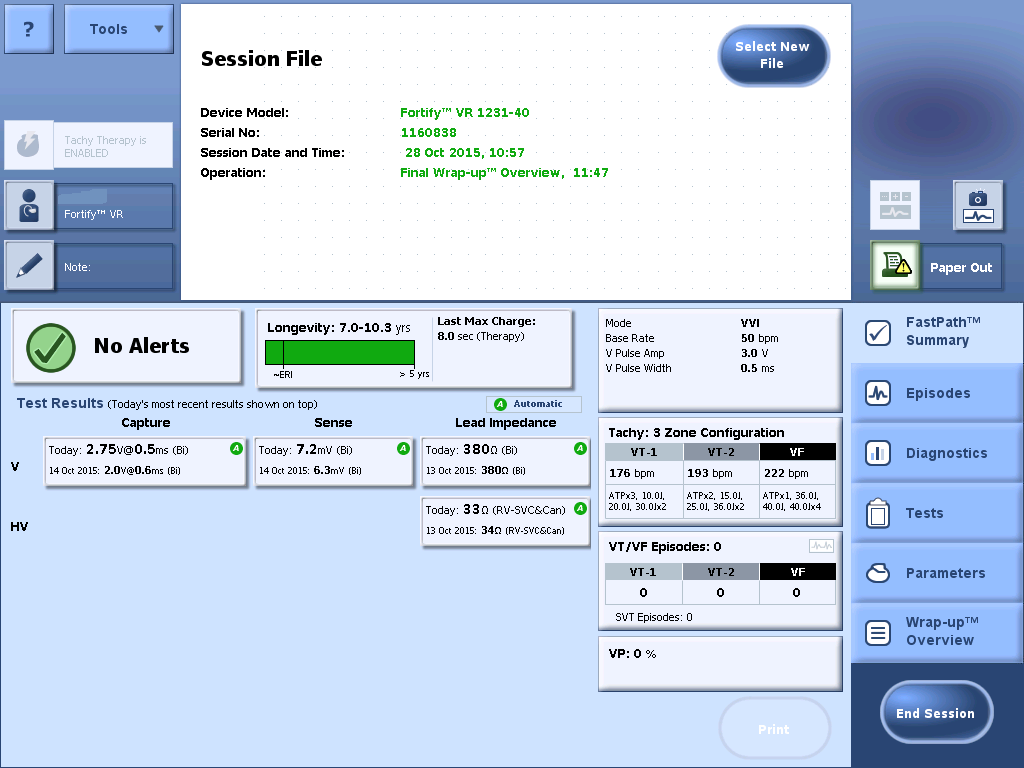


II. 16, 18, 22, 24 and 30 Nov, 2015 in 2 months post- first implant follow-up


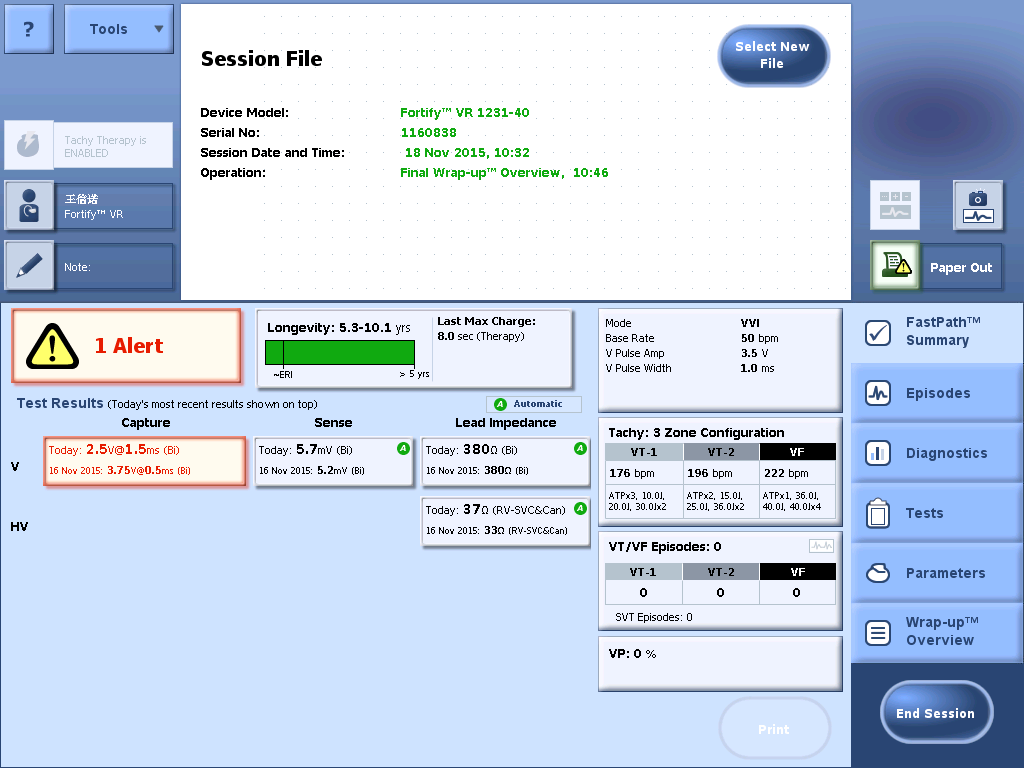


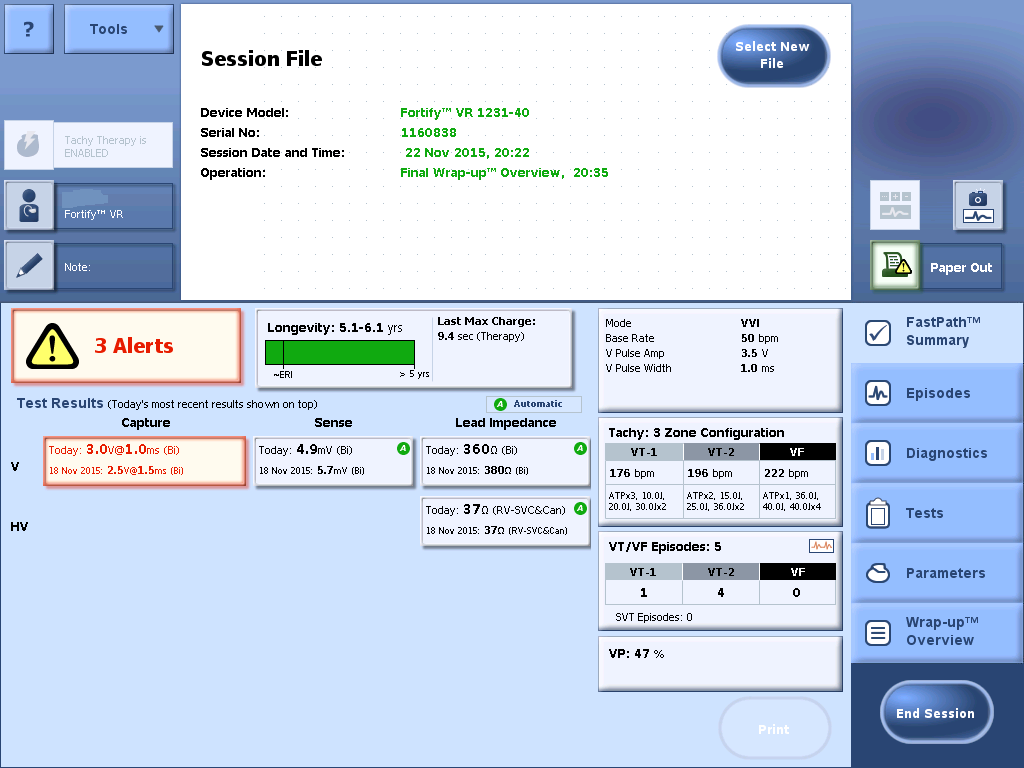


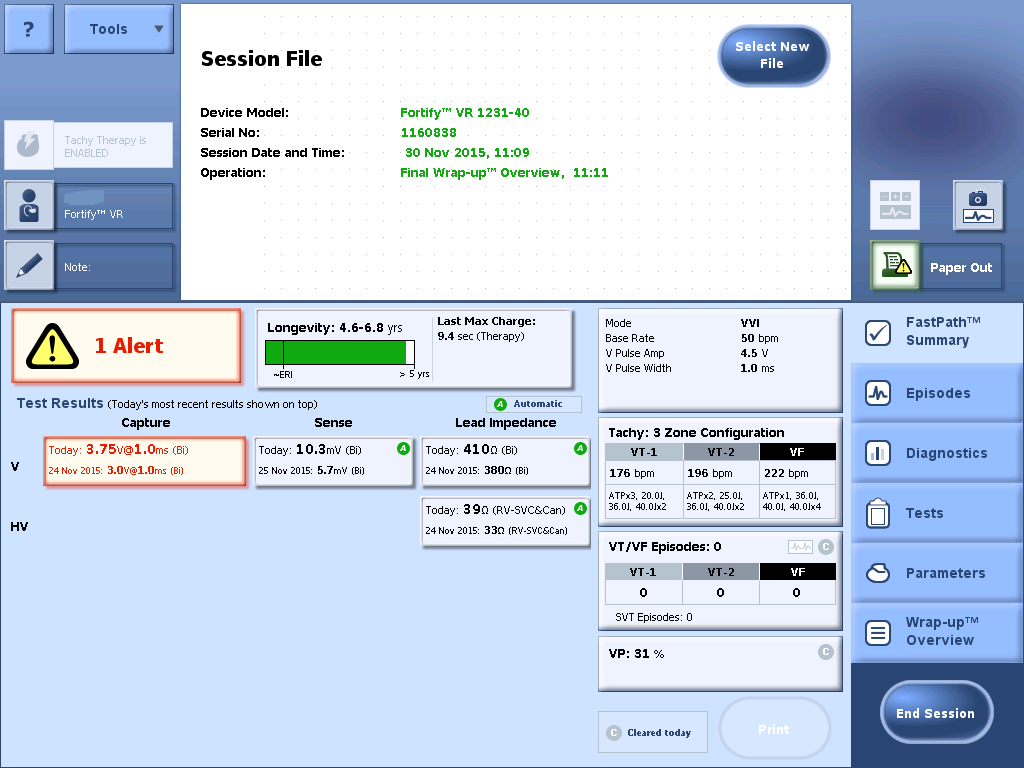


III. 1 and 3 Dec, 2015 in 2 months post- first implant follow-up


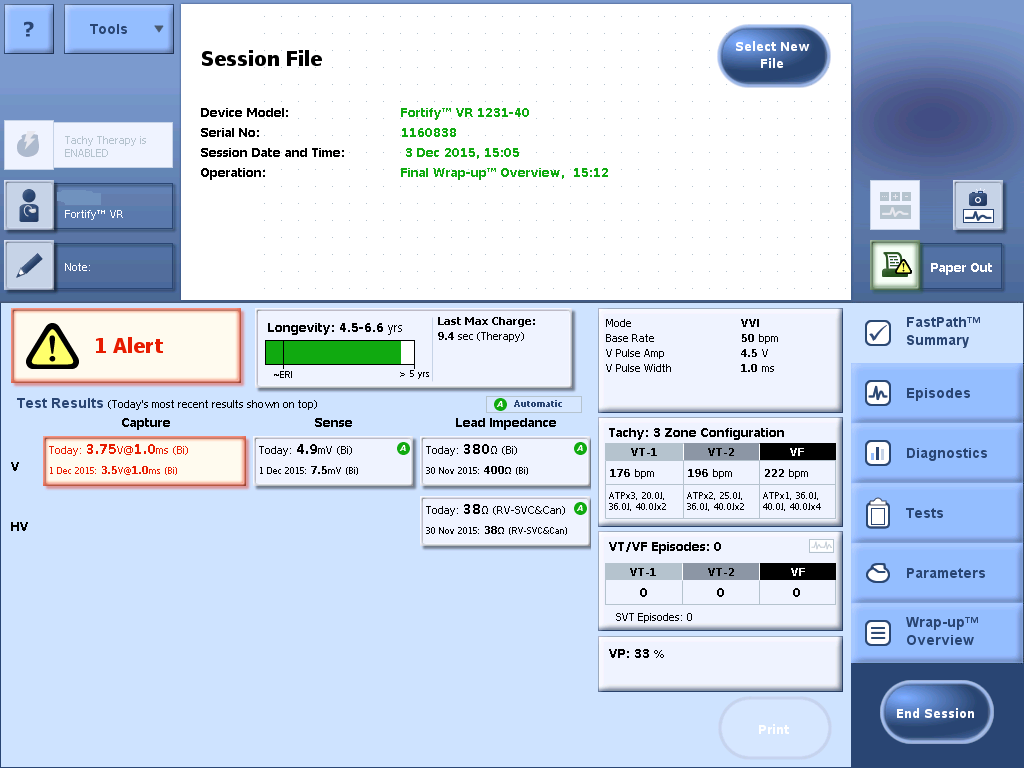


IV. 8, 9 and 16 Dec, 2015 in two weeks post- second implant follow-up


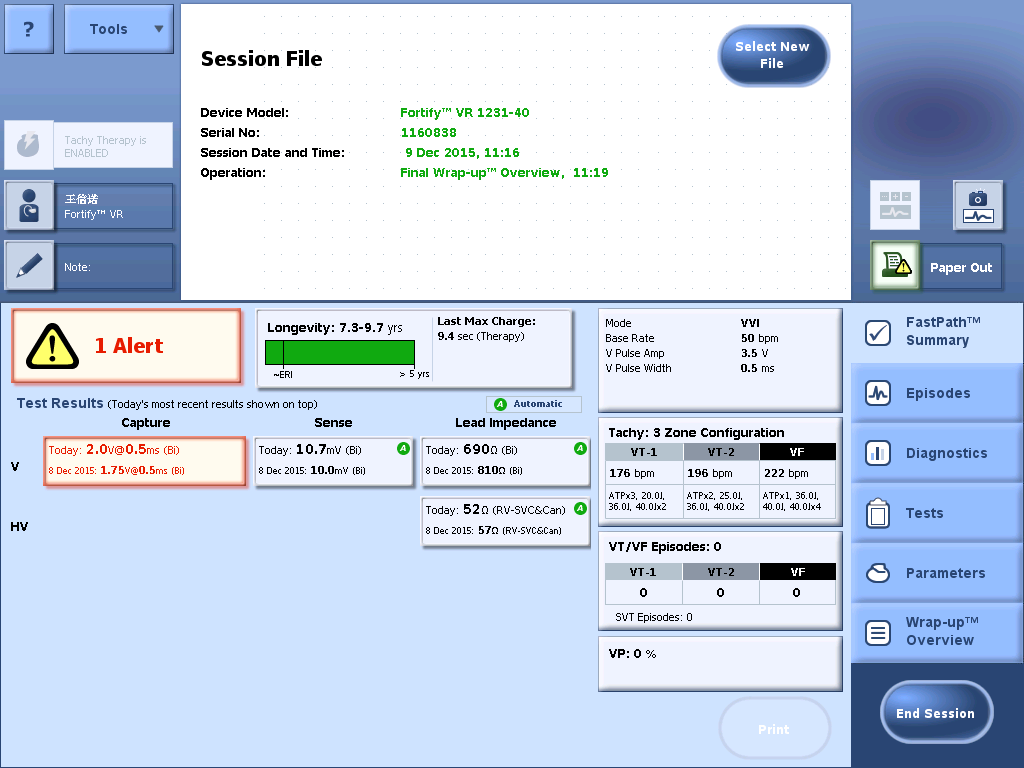


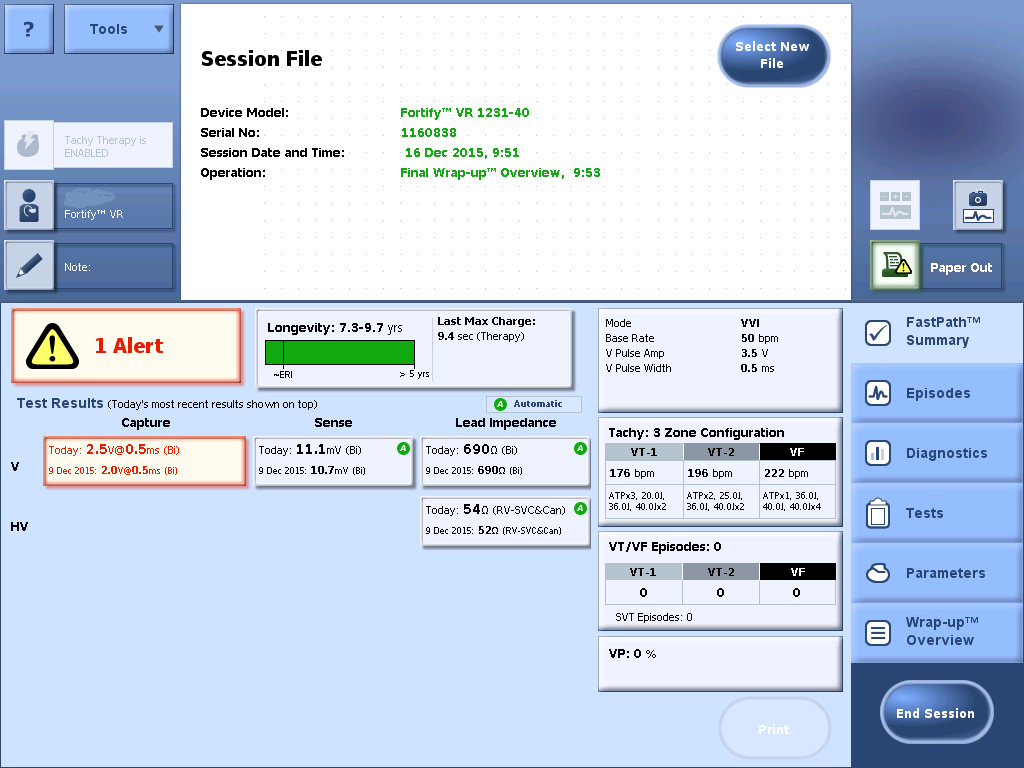


V. 9 Mar and 13 Apr, 2016 in 3-4 months post- second implant follow-up


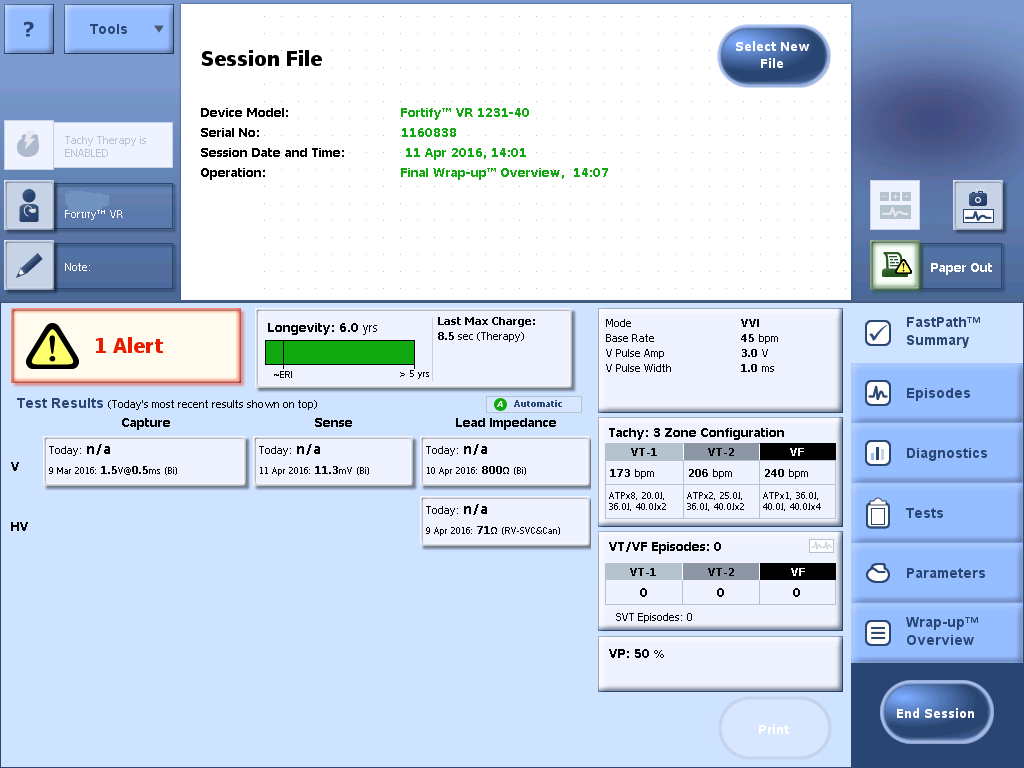


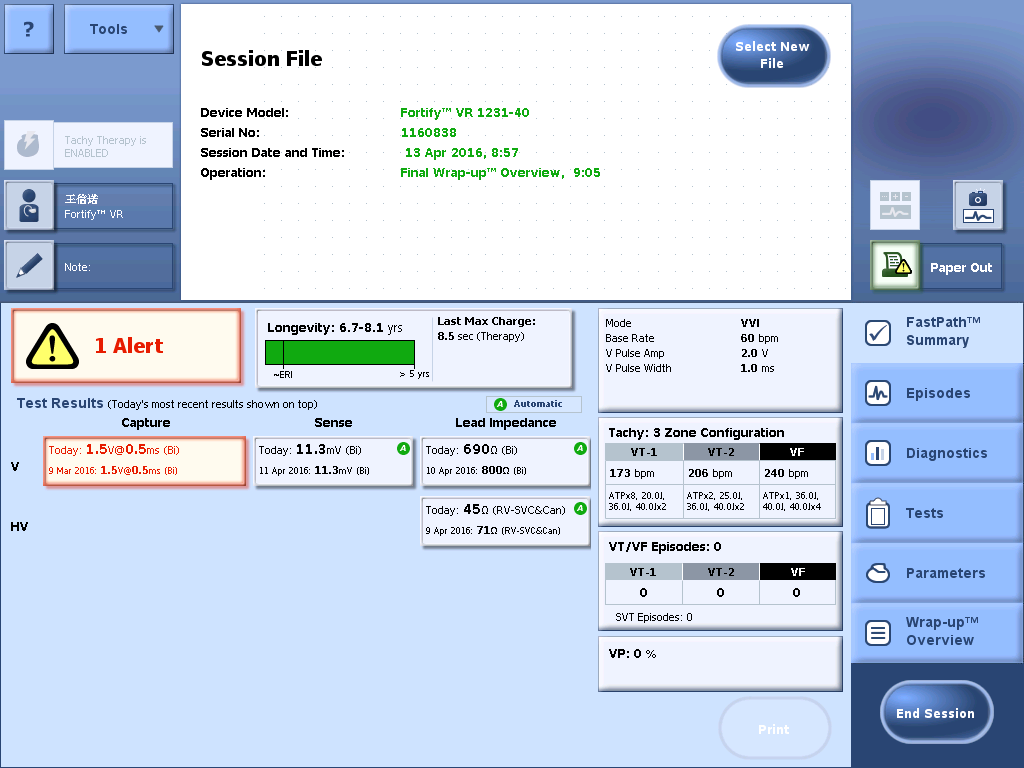


VI. 9 Nov, 2016 in 12 months post- second implant follow-up


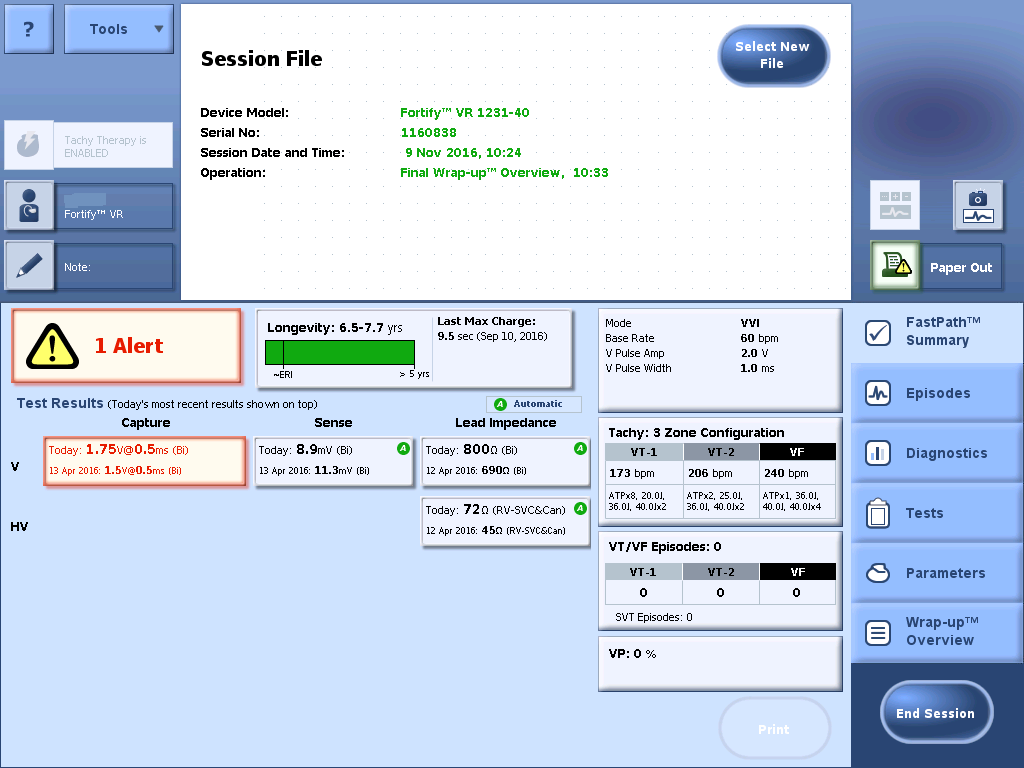


VII. 14 Jun, 2016 in 18 months post- second implant follow-up


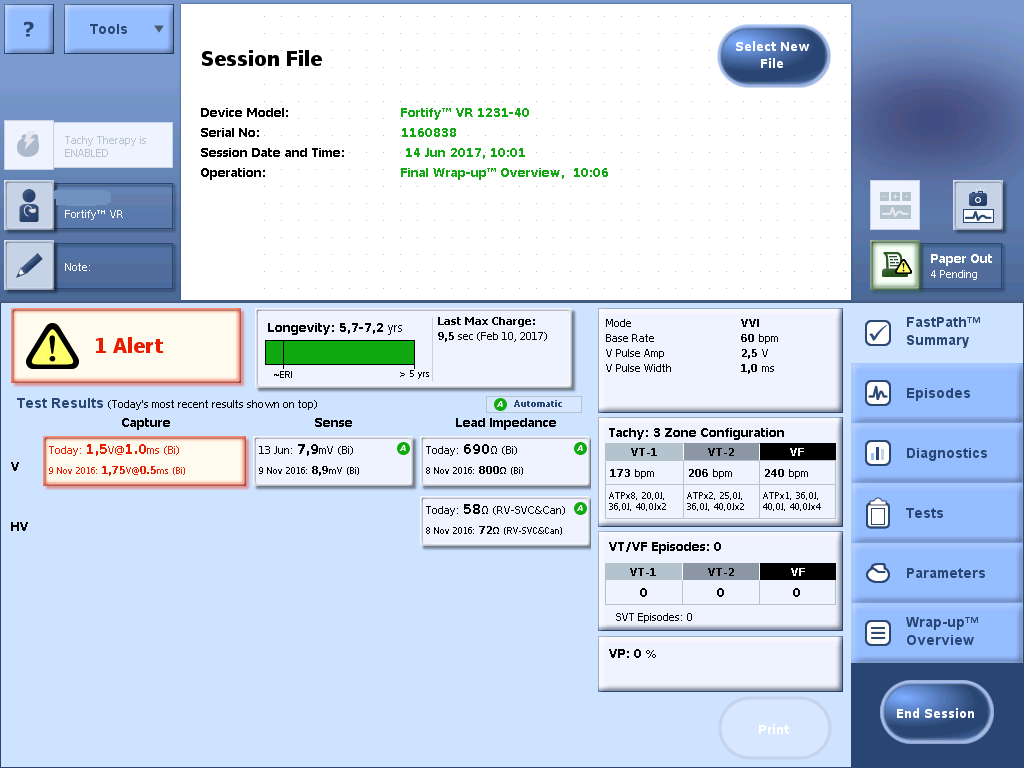


VIII. 27 Sep, 2016 in 22 months post- second implant follow-up


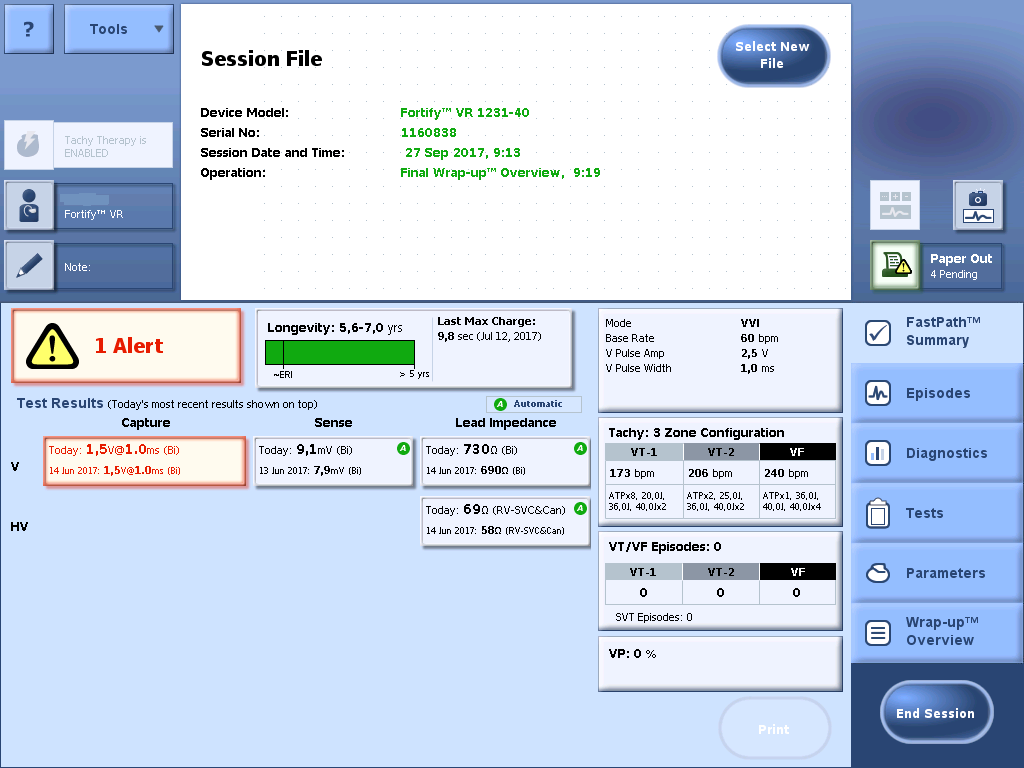

Supplement: Additional file 1: — I. 14 and 28 Oct, 2015 in 4 weeks post-first implantation follow-up. II.16, 18, 22, 24 and 30 Nov, 2015 in 2 months post- first implantation follow-up. III.1 and 3 Dec, 2015 in 2 months post- first implantation follow-up. IV.8, 9 and 16 Dec, 2015 in two weeks post- second implantation follow-up. V. 9 Mar and 13 Apr, 2016 in 3–4 months post- second implantation follow-up. VI. 9 Nov, 2016 in 9 months post- second implantation follow-up. VII. 14 Jun, 2017 in 18 months post- second implantation follow-up. VIII. 27 Sep, 2017 in 22 months post- second implantation follow-up. Initial threshold of device interrogation was respectively 2.37 V/0.5 ms, 2.75 V/0.5 ms, and 3.75 V/1.0 ms in 2, 4 and 9 weeks post- implantation. Recent device interrogation showed a ventricular sensing of 8.9 mV, 7.9 mV and 9.1 mV, a pacing threshold of 1.75 V/0.5 ms, 1.5 V/1.0 ms and 1.5 V/1.0 ms at 13, 20, 24 months post-implantation follow-up. (DOCX 902 kb) [file 12872_2017_695_MOESM1_ESM.docx]
